# Cellular Immune Function in Myalgic Encephalomyelitis/Chronic Fatigue Syndrome (ME/CFS)

**DOI:** 10.3389/fimmu.2019.00796

**Published:** 2019-04-16

**Authors:** Jacqueline M. Cliff, Elizabeth C. King, Ji-Sook Lee, Nuno Sepúlveda, Asia-Sophia Wolf, Caroline Kingdon, Erinna Bowman, Hazel M. Dockrell, Luis Nacul, Eliana Lacerda, Eleanor M. Riley

**Affiliations:** ^1^Department of Immunology and Infection, Faculty of Infectious and Tropical Diseases, London School of Hygiene & Tropical Medicine, London, United Kingdom; ^2^Centre of Statistics and Applications, University of Lisbon, Lisbon, Portugal; ^3^Department of Clinical Research, Faculty of Infectious and Tropical Diseases, London School of Hygiene & Tropical Medicine, London, United Kingdom

**Keywords:** myalgic encephalomyelitis, chronic fatigue syndrome, natural killer cells, T cell differentiation, MAIT cells, herpes viruses

## Abstract

Myalgic encephalomyelitis/chronic fatigue syndrome (ME/CFS) is a debilitating condition with unknown aetiology, Myalgic encephalomyelitis unclear pathophysiology and with no diagnostic test or biomarker available. Many patients report their ME/CFS began after an acute infection, and subsequent increased frequency of infections, such as colds or influenza, is common. These factors imply an altered immunological status exists in ME/CFS, in at least a proportion of patients, yet previous studies of peripheral immunity have been discrepant and inconclusive. The UK ME/CFS Biobank, which has collected blood samples from nearly 300 clinically-confirmed ME/CFS patients, enables large-scale studies of immunological function in phenotypically well-characterised participants. In this study, herpes virus serological status and T cell, B cell, NK cell and monocyte populations were investigated in 251 ME/CFS patients, including 54 who were severely affected, and compared with those from 107 healthy participants and with 46 patients with Multiple Sclerosis. There were no differences in seroprevalence for six human herpes viruses between ME/CFS and healthy controls, although seroprevalence for the Epstein-Barr virus was higher in multiple sclerosis patients. Contrary to previous reports, no significant differences were observed in NK cell numbers, subtype proportions or *in vitro* responsiveness between ME/CFS patients and healthy control participants. In contrast, the T cell compartment was altered in ME/CFS, with increased proportions of effector memory CD8^+^ T cells and decreased proportions of terminally differentiated effector CD8^+^ T cells. Conversely, there was a significantly increased proportion of mucosal associated invariant T cells (MAIT) cells, especially in severely affected ME/CFS patients. These abnormalities demonstrate that an altered immunological state does exist in ME/CFS, particularly in severely affected people. This may simply reflect ongoing or recent infection, or may indicate future increased susceptibility to infection. Longitudinal studies of ME/CFS patients are needed to help to determine cause and effect and thus any potential benefits of immuno-modulatory treatments for ME/CFS.

## Introduction

Myalgic encephalomyelitis (or encephalopathy)/chronic fatigue syndrome (ME/CFS) is characterised by unexplained persistent or recurrent incapacitating fatigue for over 6 months leading to substantial reductions in previous levels of occupational, educational, social and personal activities (1, 2) and, often, by moderate or severe physical disability and significant reductions in quality of life (3, 4). With population prevalence rates estimated at 0.1–0.4%, ME/CFS has considerable societal and economic impacts due to its chronic nature, its tendency to affect young adults in the most productive periods of their lives, and its impact on family and friends (3, 5–7). The aetiology remains elusive. Currently, there are no confirmatory diagnostic tests or specific, evidence-based treatments that are widely accepted and that consider the heterogeneity of symptoms and their fluctuating nature. Thus, despite some significant recent advances (8, 9), ME/CFS continues to be poorly understood. Unfortunately, many apparently interesting research findings results have not proven reproducible (10), due in part to the small size and cross-sectional nature of many studies, and to variability between studies in the research methods used and their quality.

Acute viral infections have frequently been associated with the onset of ME/CFS (9) but no specific viral aetiology has been confirmed and there is no consistent association between persistent or chronic virus infection and ME/CFS. One possibility is that people with ME/CFS (PWME) are susceptible to acute viral infections as a consequence of underlying immune dysfunction. Numerous immune abnormalities, including altered plasma cytokine profiles, abnormal T lymphocyte activation and impaired cytotoxic responses, have been described in individuals with ME/CFS (5, 7), but recent systematic reviews and observational studies have failed to identify robust and reproducible immune biomarkers of disease or disease severity (11–13).

There have been a number of reports of inconsistent abnormalities in natural killer (NK) cell function. NK cells are large granular lymphocytes that function as innate immune effectors that, by cytokine production or cytotoxicity, contain infections (or neoplasias) until an effective adaptive response can be mounted. Individuals with inherited NK cell disorders are highly susceptible to systemic herpes virus and other opportunistic infections (14). Direct NK cell activation follows interaction with cells which lack major histocompatibility complex (MHC) Class I ligands for inhibitory NK cell receptors and/or express stress-induced ligands for NK cell activating receptors. Indirect NK cell activation occurs following microbial ligation of pattern recognition receptors on myeloid accessory cells and is mediated by cytokines (IL-12, IL-15, IL-18, IFN-α) and by contact-dependent stimuli (15, 16). PWME have been variously reported to have increased (17, 18), decreased (19, 20) or normal (21–23) numbers of circulating NK (CD16^+^ or CD56^+^, CD3^−^) cells; their NK cells are reportedly deficient in Maher et al. (24) or have enhanced expression of Huth et al. (25) the cytotoxic molecule perforin; are less able (or fully able) to lyse MHC Class 1 deficient target cells (19, 21, 22, 26); and are defective (or not) in their upregulation of activation markers after *in vitro* stimulation (21, 22, 27). Again, the reproducibility of many of these studies is hampered by their relatively small size, the diverse clinical presentations of the cases, or the limited extent of the immunological characterisation in any one study.

Importantly, only one (23) of these immunological studies has taken account of the prevalence of human cytomegalovirus (CMV) infection in cases and controls. CMV infection leaves a permanent footprint on the immune system including oligoclonal expansions and terminal differentiation of CD8^+^ T cells and expansion of a subset of highly differentiated NKG2C^+^ NK cells (28); this NK population is further expanded by subsequent viral infection (28, 29). It remains possible therefore, that the reported differences in T cell and NK cell phenotype and functional capacity between PWME and healthy controls may result from differences in the prevalence of immunomodulatory viruses such as CMV. Here we report an in-depth analysis of peripheral blood leucocyte phenotype and function in a clinically well-defined cohort of moderately and severely affected ME/CFS cases compared to non-fatigued healthy controls and, as a control for reduced levels of physical activity, people with multiple sclerosis. All participants were screened for serological evidence of human cytomegalovirus (CMV), Epstein–Barr virus (EBV), herpes simplex virus 1 (HSV1), Herpes simplex virus 2 (HSV2), varicella-zoster virus (VZV), and human herpesvirus (HHV6) infections.

## Materials and Methods

### Recruitment and Clinical Evaluation

Study participants, including PWME, multiple sclerosis (MS) and non-fatigued healthy controls, were recruited through the UK National Health Service (NHS) primary and secondary health care services. In addition, some people with clinically confirmed severe ME/CFS were identified via support groups and were invited to participate. All potential participants were rigorously assessed to ensure that they met the study case definitions for ME/CFS. Non-fatigued healthy controls were also recruited by advertisement within Higher Education Institutions or were friends or family members of PWME. Ethical approval was granted by the London School of Hygiene & Tropical Medicine (LSHTM) Ethics Committee (Ref. 6123) and the National Research Ethics Service (NRES) London-Bloomsbury Research Ethics Committee (REC ref. 11/10/1760, IRAS ID: 77765). All participants provided written informed consent for questionnaire, clinical measurement and laboratory test data, and for samples to be made available for ethically-approved research, after receiving an extensive information sheet and consent form, which included an option to withdraw from the study at any time.

All participants with ME/CFS or MS had previously received a confirmed medical diagnosis. Participants were aged between 18 and 60 years. PWME were reassessed by clinical research staff for compliance with the Canadian Consensus (2) and/or CDC-1994 (“Fukuda”) (1) criteria, which were the study case definitions, before recruitment into this study. Participants were excluded if they had (i) taken antiviral medication or drugs known to alter immune function in the preceding 3 months; (ii) had any vaccinations in the preceding 3 months; (iii) had a history of acute and chronic infectious diseases such as hepatitis B and C, tuberculosis, HIV (but not herpes virus or other retrovirus infection); (iv) another chronic disease such as cancer, coronary heart disease, or uncontrolled diabetes; (v) a severe mood disorder; (vi) been pregnant or breastfeeding in the preceding 12 months; or (vii) were morbidly obese (BMI ≥ 40). Once eligibility was confirmed, participants were invited to a recruiting centre for clinical assessment and blood sample collection: this included the healthy controls, PWME and people with MS. Those participants with severe disease and mobility restrictions were visited at home by a research nurse. Supplementary Table S1 shows the range of clinical variables including the Fatigue Severity Scale (30) collected from the study population, some of which were used for assessing eligibility. Participants were not excluded on the basis of minor ailments such as sore throat, fever/chills, tender glands, flu symptoms, and or new sensitivity/allergy, to avoid developing an artificial control group. All participants were part of the UK ME/CFS Biobank (UKMEB); further details on the method used for selection and recruitment of participants and processing of data and samples were described previously (31).

#### Power Calculation

We recruited 404 participants comprising 251 with ME/CFS (54 severely affected and 197 mild/moderate cases), 46 cases of MS, and 107 healthy controls. Based on our own data on NK cell responses to cytokine stimulation in HCMV-infected healthy adults (32) we estimated that the proposed sample size would provide at least 80% power to detect a 30% difference in NK cell responses between MS and severely affected cases (the comparison requiring the highest sample size) and >90% power to detect a 20% difference in NK cell response between cases and healthy controls (Epi Info, version 3.5.53).

### Sample Collection and Processing, Including PBMC Freezing

Blood samples were collected in sodium heparin (for PBMC) or in potassium EDTA (for plasma) vacutainers (Becton Dickinson) and transported within Pathopak containers (in compliance with UN 3373 regulations), at room temperature (18–25°C) protected from direct sunlight and extremes of environmental temperature. Samples were delivered to the University College London / Royal Free Hospital (UCL/RFH) BioBank laboratories for processing and storage within 6 h of collection: the average time from collection to processing was 2.5 h across all four study groups. Serum, plasma, and peripheral blood mononuclear cells (PBMC) were processed and aliquoted in volumes of 200 μl to 2 ml. PBMCs were aliquoted at 5 × 10^6^ cells/ml. Aliquots were stored in 1 or 2 ml cryotubes, in vapour phase liquid nitrogen at −180°C, and shipped to LSHTM for laboratory analysis on dry ice. Each aliquot was labelled with a 2D bar code corresponding to a unique 15 digit ISBT 128 (global standard) identifier which also served to anonymise the samples. All laboratory evaluations were performed in a blinded manner with samples identified only by their unique identifier.

### Herpesviruses Serology

Plasma concentrations of IgG to herpes simplex virus-1 (HSV 1), HSV 2, Varicella Zoster virus (VZV), human cytomegalovirus (CMV), Epstein Barr virus (EBV) viral capsid antigen (VCA), and EBV nuclear antigen 1 (EBNA 1) and human herpes virus 6 (HHV 6) were assayed by commercial quantitative ELISA [Demeditec Diagnostics, Germany for all viruses, except HHV6 assay from VIDIA (Vestec, Czech Republic)] according to the manufacturer's instructions. IgG concentrations were expressed as arbitrary units per millilitre (U/ml). For HSV1, HSV 2, VZV, CMV and EBV, samples with IgG concentration ≤8 U/ml were scored as negative and those with concentration ≥12 U/ml were regarded as positive. Samples with IgG concentration between 8 and 12 were regarded as equivocal and were re-tested. Samples were recorded as EBV seropositive if they gave a positive reaction to EBV-VCA and/or EBNA 1. For HHV-6, concentrations ≤10.5 U/ml were considered negative, while concentrations ≥12.5 U/ml were considered positive. Samples giving concentrations between 10.5 and 12.5 U/ml were considered equivocal and re-tested.

### Leucocyte Phenotyping

Cryopreserved PBMCs were transported to the research laboratory on dry ice, and analysed in mixed batches containing samples across the four clinical groups. PBMC were thawed by addition of warm RPMI-1640 tissue culture medium (Invitrogen), washed in RPMI containing 1% foetal bovine serum (Invitrogen) and counted by trypan blue exclusion. Cells were resuspended at 4 x 10^6^ cells/ml in complete medium [RPMI-1640 plus 10% human AB serum (Sigma) and 1% penicillin, streptomycin/glutamine (Invitrogen)]. Cells were either used immediately for *ex vivo* phenotyping or were placed in culture for functional analysis. For flow cytometry, cells (200,000/well) were aliquoted into 96 well V-bottom microtitre plates, stained as described previously (32) in Cytofix/Cytoperm™ for intracellular stains, and analysed on an LSR-II flow cytometer (Becton Dickinson). Antibody staining panels are shown in Supplementary Tables S2, S3. Data were collected in FACSDiva v6.1 and analysed using Flowjo v10 (TreeStar) and SPICE (33) software.

### Functional Assays

Cells were aliquoted (200,000 cells/well) into U-bottom microtitre plates together with one of the following stimuli: complete medium (negative control), cell stimulation cocktail (81 nM PMA/1.34 μM ionomycin; eBioscience); CpG2216 (2.5 μM; Invivogen), IL-12+IL-18 (5 and 50 ng/ml respectively; Peprotech and R&D, respectively) or MHC Class-I deficient K562 cells (at a host:target cell ratio of 2:1). In addition, cells were plated into flat-bottom microtitre plates that had previously been coated overnight with anti-CD16 antibody (20 μg/ml, Becton Dickinson) or an isotype control antibody (20 μg/ml; eBioscience). After incubation at 37°C in 5% CO_2_ for 1 h (cell stimulation cocktail) or 15 h (all other stimuli), brefeldin A (Golgiplug; final concentration 1:1,000; Becton Dickinson) and monensin (Golgistop, final concentration 1:1,500; Becton Dickinson) were added to each well and plates were then incubated for a further 3 h before staining for flow cytometry (as above). For any assays of CD107a expression, anti-CD107a antibody was added to wells at the start of the culture period. The 17 h incubation period was selected, based on our previous reports (34–36), to allow for upregulation of both IFNγ and CD25, and degranulation via both direct and indirect mechanisms.

### Data Analysis

The primary data analysis comprised four groups of participants: people with mild/moderate ME/CFS (ME-M), people severely affected by ME/CFS (ME-S), healthy controls (C), and people with multiple sclerosis (MS). All data analyses were carried out in a blinded manner with groups identified by a code number; the code was broken only after all analyses were complete.

Univariate analyses were conducted using GraphPad Prism v7.01 unless otherwise stated. Continuous variable datasets were checked for Normal distribution by D'Agostino & Pearson test, and analysed by ANOVA or Kruskal-Wallis test for parametric or non-parametric datasets respectively when comparing clinical groups, or by Student's *T*-test or Mann Whitney test respectively when comparing the CMV serostatus on the outcome variables. All *P*-values reported are after correcting for multiple testing using Tukey's test for parametric data or Dunn's test for non-parametric data. Differences between clinical groups in herpesvirus seroprevalences were analysed by Chi squared test, using Stata version 15.1. Receiver Operating Characteristic (ROC) curve analysis was conducted in GraphPad Prism.

The above analysis was extended to control for possible confounding variables using R statistical software (37). For flow cytometry data, the percentages of each cell subpopulation were conveniently transformed into the respective log-odds [e.g., log %CD4^+^/(100%–%CD4^+^)]: the resulting data followed approximately normal distributions. Multiple linear regression was used to compare the outcome variables between groups; all regression models included gender, age at recruitment, analysis batch and seropositivity for each herpes virus as confounding factors. To determine the significance of differences between clinical groups, regression models including group as a variable were compared with models excluding group using the likelihood ratio test. The significance level of each individual test was determined to ensure an overall significance level in a context of multiple testing. The significance level was pre-specified at 5% for each separate analysis.

## Results

### Study Participant Characteristics

The final cohort comprised 251 people with ME/CFS (197 mild/moderate and 54 severe), 46 people with MS and 107 healthy controls. The demographic and clinical characteristics of the cohort are summarised in Table 1. Symptoms typically associated with infection or inflammation were more frequent and more severe among PWME than among those with MS, although mild symptoms of inflammation were present in nearly 50% of people with MS. Most healthy controls did not present with any symptoms (*P* < 0.0001). Fatigue severity scores were, as expected, higher in PWME, with a trend towards higher scores in those severely affected, followed by people with mild/moderate ME/CFS, then people with MS and healthy controls presenting very low levels of fatigue (*P* < 0.001). Erythrocyte Sedimentation Rate (ESR), a non-specific marker of inflammation, was slightly raised in people with mild/moderate ME/CFS, with similar median values found in all other groups (*P* = 0.01). Those with MS were slightly older compared to other study groups, but distribution by sex, deprivation index, and total white blood cells and lymphocytes was similar across the groups.

Table 1Description of study population, in relation to the following variables–demographic, socio-economic, immune-related symptoms, Fatigue Severity Scores, viral serology, and baseline routine blood tests results.

**Table:** 

**Discrete variables**	**Category of recruitment**								**Total**		*P*-value^*^
	**ME/CFSsa**		**ME/CFSmm**		**MS**		**HC**			
	**(*n*)**	**(%)**	**(*n*)**	**(%)**	**(*n*)**	**(%)**	**(*n*)**	**(%)**	**(*n*)**	**(%)**	
**SEX**											
Male	13	24.1	47	24.0	10	20.4	28	26.2	98	24.1	0.89
Female	41	75.9	149	76.0	39	79.6	79	73.8	308	75.9	
**IMMUNE-RELATED SYMPTOMS**											
No symptoms	0	0.0	10	5.1	25	51.0	74	69.1	109	26.9	0.000
Some symptoms	22	40.7	83	42.6	20	40.8	31	29.0	156	38.5	
Moderate/severe symptoms	32	59.3	102	52.3	4	8.2	2	1.9	140	34.6	
**VIRAL SEROLOGY RESULTS**											
CMV positive	18	33.3	57	29.4	18	45.0	40	37.4	133	33.8	0.40
EBV combined positive	48	88.9	171	88.1	39	95.5	99	93.4	357	90.6	0.42
HSV1 positive	29	53.7	82	42.3	23	57.5	45	42.4	179	45.4	0.49
HSV2 positive	22	40.7	76	39.2	16	40.0	36	33.9	150	38.1	0.12
VZV positive	52	96.3	190	97.9	39	97.5	104	98.1	385	97.7	0.85
HHV6 positive	52	96.3	177	91.2	36	90.0	102	96.2	367	93.1	0.48

**Table:** 

**Continuous variables**	**ME/CFSsa**		**ME/CFSmm**		**MS**		**HC**		***P***-value^*^
	**Median**	**IQR**	**Median**	**IQR**	**Median**	**IQR**	**Median**	**IQR**	
Age (years)	46	32–51	43	34–52	51	32–52	44	32–52	0.002
Index of multiple deprivation (ranking)	18,883	12,463–24,921	16,873	11,177 −23,373	15,853	12,567–21,921	17,02	13,564–25,988	0.29
Fatigue severity scale scores	6.67	6.3–6.89	6.55	6.00–7.00	5.61	3.67–6.44	2.17	1.55–2.67	0.000
**BASELINE BLOOD TESTS RESULTS**									
White blood cells (10^9^/L)	5.88	5.13–7.65	6.10	5.20–6.93	6.01	5.10–7.30	5.99	5.12–6.86	0.93
Lymphocytes (10^9^/L)	1.78	1.57–2.03	1.91	1.60–2.31	1.81	1.49–2.15	1.82	1.52–2.21	0.15
ESR (mm/h)	5	2–10	7	5–12	5	2–8	5	2–8	0.012

*ESR, Erythrocyte sedimentation rate; CMV, Cytomegalovirus; EBV, Epstein–Barr virus [combined results for Viral capsid antigen (VCA) and Epstein-Barr nuclear antigen (EBNA)]; HSV1, Herpes simplex virus 1; HSV2, Herpes simplex virus 2; VZV, varicella-zoster virus; HHV6, Human herpesvirus 6; ME/CFSsa, people with ME/CFS with severe clinical manifestations; ME/CFSmm, people with ME/CFS with mild/moderate manifestations; MS, people with multiple sclerosis; HC, people recruited as healthy controls*.

* *P-values—from χ^2^ tests for categorical variables and Kruskal-Wallis test for continuous variables (which were non-normally distributed)*.

### Serology

Plasma samples were tested by quantitative ELISA for six of the human herpesviruses, HSV-1, HSV-2, VZV, CMV, EBV, and HHV6. There were no significant differences between the groups in the proportions of individuals who were seropositive for any of these viruses (Table 1). However, concentrations of anti-EBV VCA varied significantly among the groups (Kruskal-Wallis test: *P* = 0.016) and this was due to higher concentrations of anti-EBV VCA antibodies among people with MS (Supplementary Figure S1).

### Leucocyte Phenotyping

**Figure 1:**
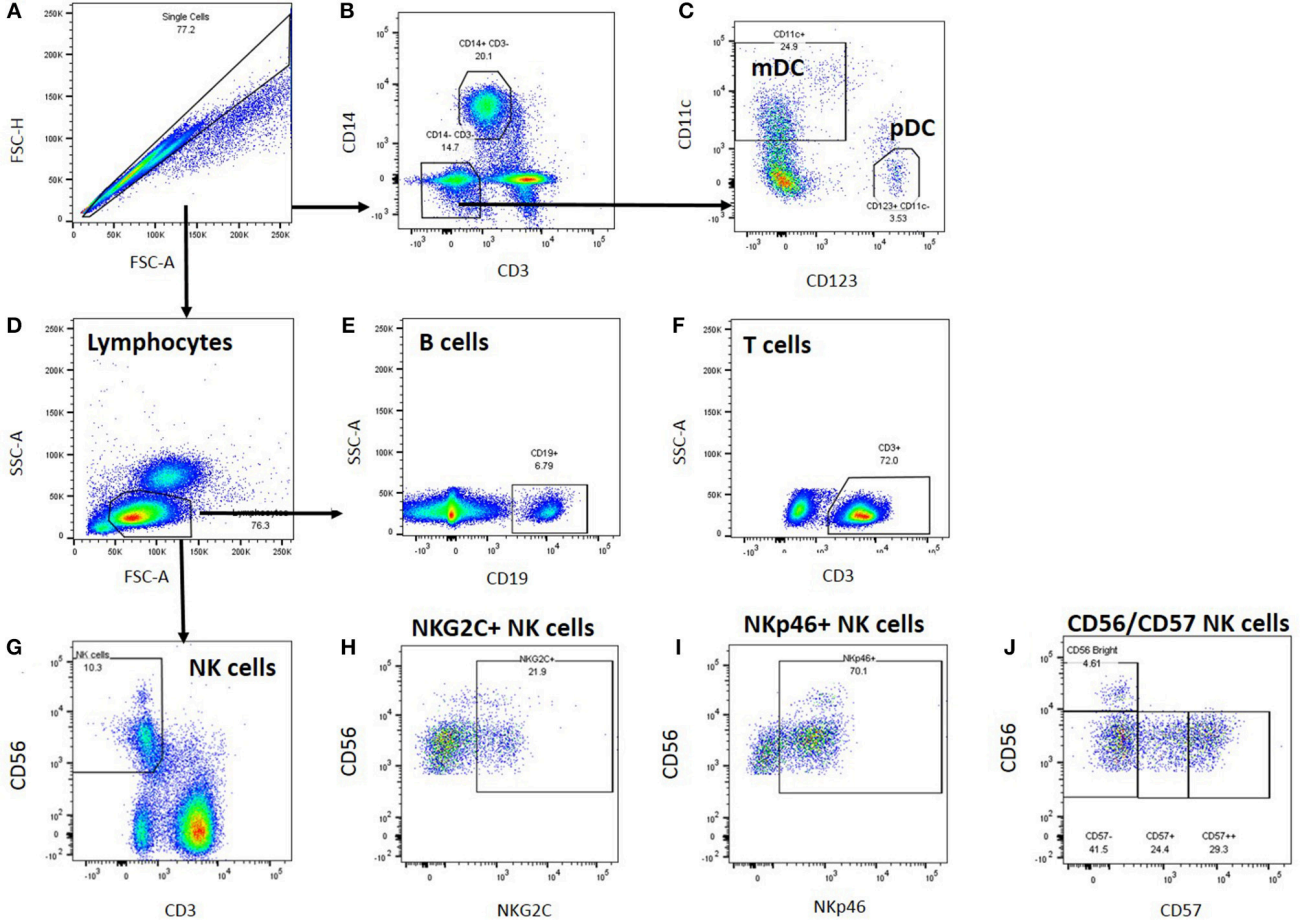
Leucocyte gating strategy for phenotypic characterisation of PBMC. Thawed PBMC from Biobank participants were stained with labelled antibodies (Supplementary Table S2), expression data were collected by Flow cytometry and analysed using FlowJo. **(A)** Single cells were gated based on their forward scatter height and area, and **(B)** monocytes within the single cell gate were identified as CD14^+^. **(C)** Dendritic cells were identified within the CD3-CD14- cell population as myeloid (CD11c+) or plasmacytoid (CD123^+^) DCs. **(D)** Lymphocytes were identified based on their forward and side scatter area profiles, and B cells were identified as CD19^+^ **(E)**, T cells as CD3^+^ **(F)**, and NK cells as CD56^+^ **(G)**. Within the NK cell gate, NK cells were characterised further based on NKG2C **(H)**, NKp46 **(I)**, and CD57 **(J)** co-expression.

Cryopreserved peripheral blood mononuclear cells were thawed and analysed by flow cytometry; cell gating is shown in Figure 1. A generous leucocyte gate was set to include all live cells and then a more restrictive lymphocyte gate was set. Within the leucocyte gate, monocytes were identified by CD14 positivity and CD3 negativity whilst dendritic cells were identified as CD14/CD3 negative and either CD11c positive (myeloid DC) or CD123 positive (plasmacytoid DC). Within the lymphocyte gate, T cells were gated as CD3^+^ and either CD4^+^ or CD8^+^; B cells as CD19^+^ and natural killer (NK) cells as CD3 negative/CD56 positive. NK cells were subsequently analysed for expression of NKG2C, NKp46, and CD57.

**Figure 2:**
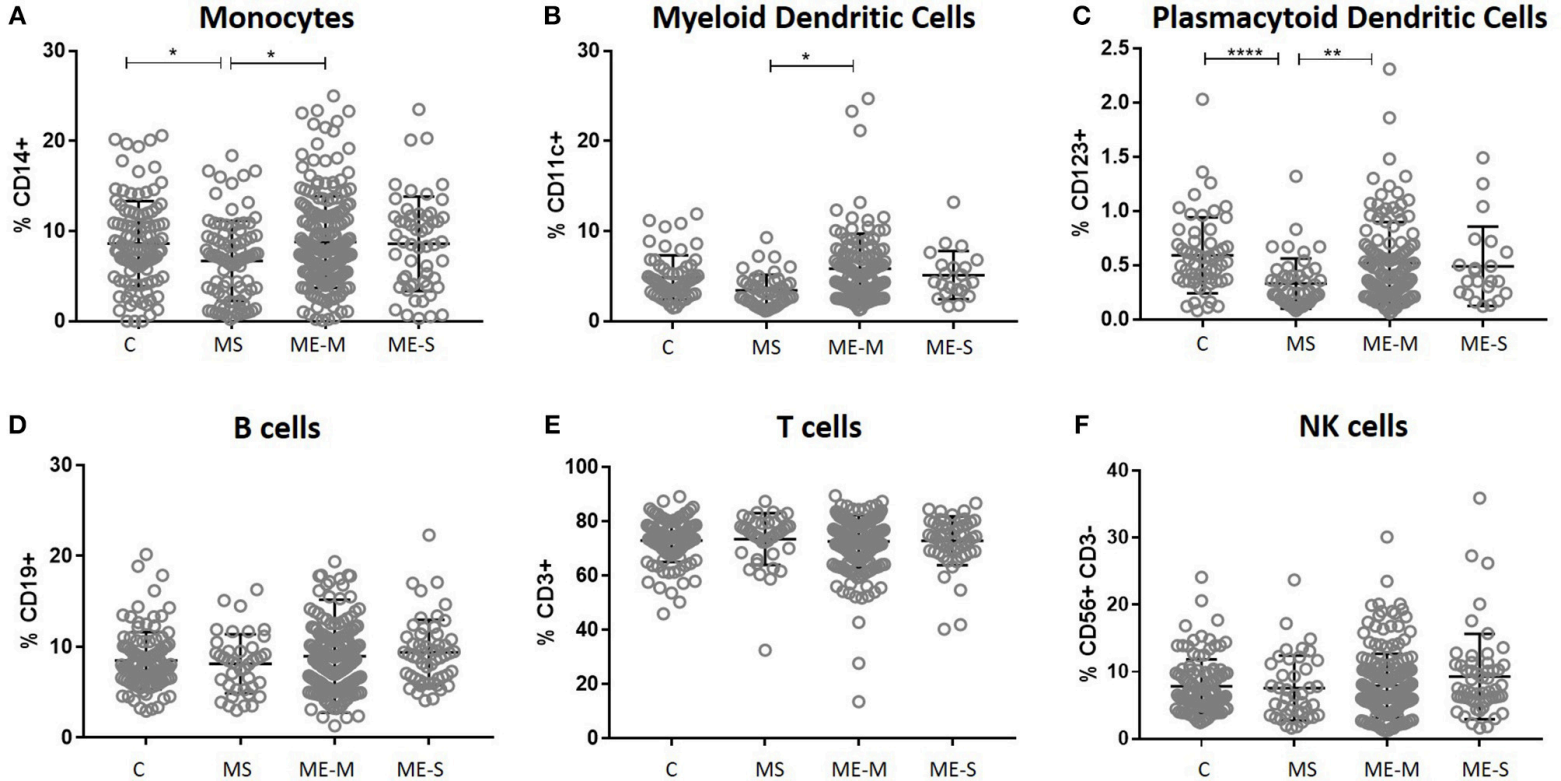
Leucocyte populations in peripheral blood from people with ME/CFS, people with MS and healthy controls. PBMC were analysed by flow cytometry, as described in Figure 1. The proportions of PBMC which were **(A)** monocytes, **(B)** myeloid dendritic cells, **(C)** plasmacytoid dendritic cells, **(D)** B cells, **(E)** T cells or **(F)** NK cells were calculated and shown for individual study participants. The bars show the Mean ± SD. Data are from healthy controls (C, *n* = 107), multiple sclerosis (MS: *n* = 46), mild/moderate ME/CFS (ME-M: *n* = 197), and severely affected ME/CFS (ME-S: *n* = 54) for **(A,D–F)** and from C (*n* = 56), MS (*n* = 46), ME-M (*n* = 120), and ME-S (*n* = 21) for **(B,C)**. Clinical groups were compared by Kruskal-Wallis test for non-parametric data: ^*^P < 0.05, ^**^P < 0.01, ^****^P < 0.0001.

In an unadjusted ANOVA analysis, there were significant differences between the groups in the proportions of live leucocytes that were monocytes (*P* = 0.0133), myeloid DCs (*P* < 0.0121), and plasmacytoid DCs (*P* < 0.0001) (Figure 2): these differences were due to altered frequencies in the multiple sclerosis patients, and disappeared after adjusting for gender, age and herpesvirus sero-positivity (not shown). By contrast, the proportions of leucocytes that were B cells, T cells or NK cells did not differ significantly among the groups in either the adjusted or the unadjusted analysis.

**Figure 3:**
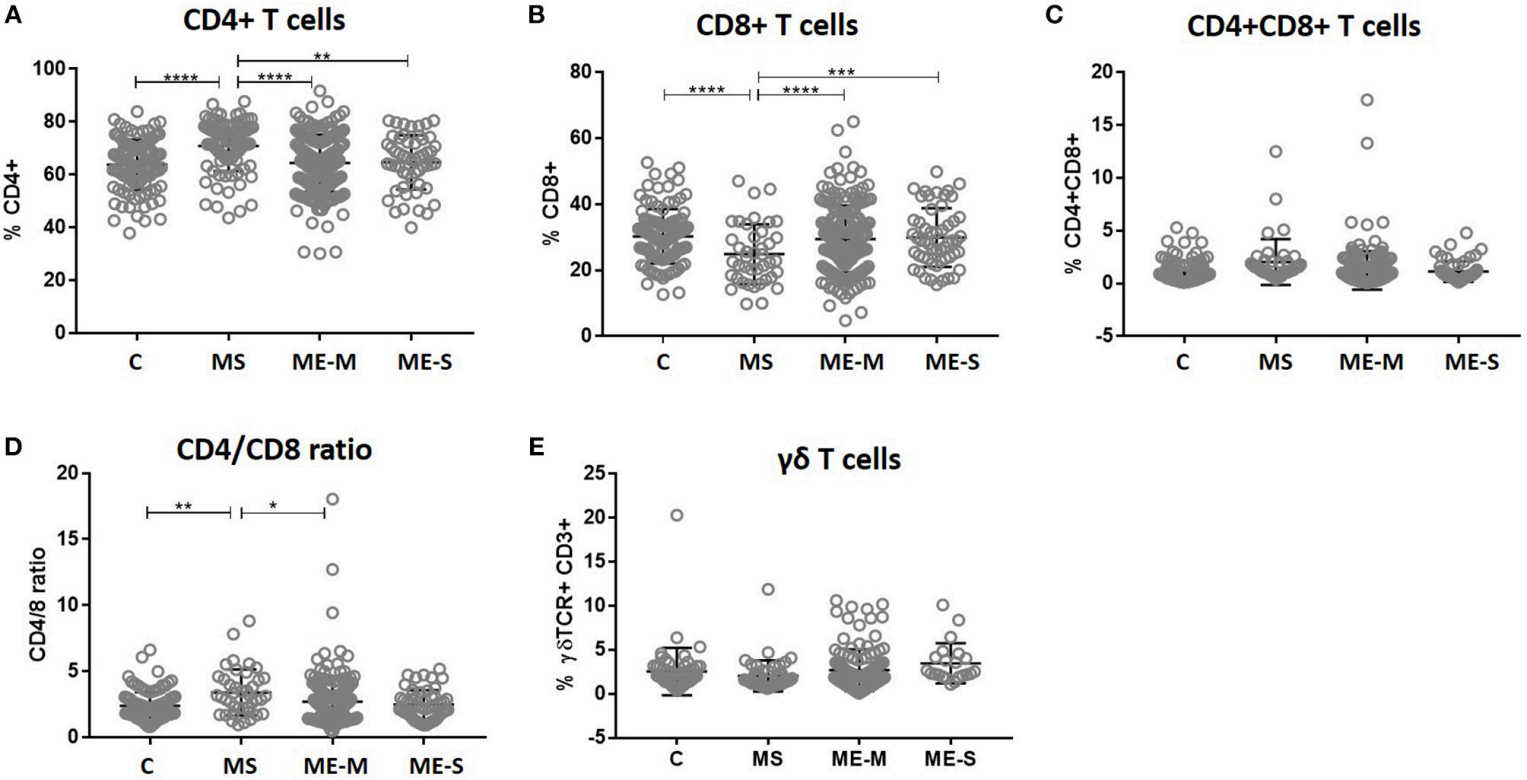
T cell subset quantification in PBMC from people with ME/CFS, people with MS and healthy controls. Within the T cell gate (CD3^+^), the CD4^+^ and CD8 staining was characterised to calculate the proportion of **(A)** CD4+ T cells, **(B)** CD8^+^ T cells, **(C)** double positive CD4^+^CD8^+^ T cells, and **(D)** the ratio of CD4^+^:CD8^+^ T cells. **(E)** The proportion of T cells which expressed the γδ TCR within the CD3+ T cell population were determined. Data are from healthy controls (C: *n* = 107), multiple sclerosis (MS: *n* = 46), mild/moderate ME/CFS (ME-M: *n* = 197), and severely affected ME/CFS (ME-S: *n* = 54) for **(A–D)**, and from C (*n* = 56), MS (*n* = 46), ME-M (*n* = 120), and ME-S (*n* = 21) for **(E)**. Clinical groups were compared by Kruskal-Wallis test for non-parametric data: ^*^P < 0.05, ^**^P < 0.01, ^***^P < 0.001, ^****^P < 0.0001.

Within the lymphocyte gate, the proportions of cells that were either CD4^+^ T cells or CD8^+^ T cells (and, as a consequence, the CD4/CD8 ratio) differed significantly between groups in the unadjusted analysis (ANOVA *P* < 0.0001 in each case) and these differences persisted after adjusting for confounders (*P* < 0.0001 in each case) (Figure 3). However, these differences were entirely due to significantly higher proportions of CD4^+^ T cells, lower proportions of CD8^+^ T cells and thus a higher CD4/CD8 ratio in the MS patients; there were no significant differences in lymphocyte distribution between ME/CFS cases and healthy controls. Similarly, there were no significant differences between the groups in the absolute numbers of different leucocyte and lymphocyte populations (Supplementary Figure S2).

**Figure 4:**
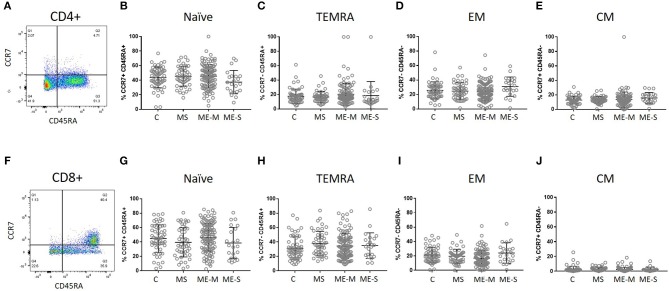
Delineation of T cell naïve and memory cells in ME/CFS and MS patients and controls. CD4^+^ **(A–E)** and CD8^+^ **(F–J)** T cells were analysed for CD45RA and CCR7 co-expression to quantify proportions which were naïve CD45RA^+^CCR7^+^ **(B,G)**, effector memory RA (TEMRA) CD45RA^+^CCR7^−^ **(C,H)**, effector memory CD45RA^−^CCR7^−^ **(D,I)** or central memory CD45RA^−^CCR7^+^ T cells. The bars show the Mean and SD. Data are from C (*n* = 56), MS (*n* = 46), ME-M (*n* = 120), and ME-S (*n* = 21). Clinical groups were compared by Kruskal-Wallis test for non-parametric data.

**Figure 5:**
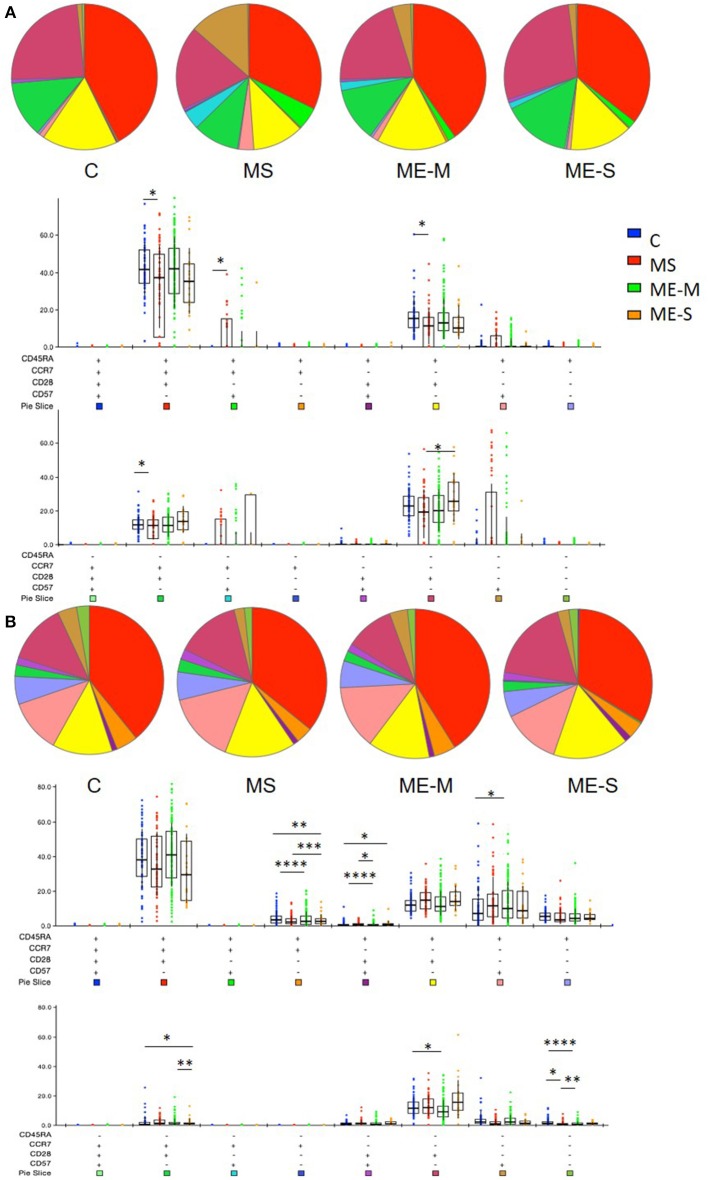
Proportions of differentiated T cell populations in people with ME/CFS, people with MS and healthy controls. CD4^+^ **(A)** and CD8^+^ **(B)** T cells were analysed for CCR7, CD45RA, CD28, and CD57 co-expression to quantify proportions of T cells expressing combinations of these markers using FlowJo and SPICE software. The pie charts show the median proportions of each cell type in each clinical group. In the bar and whisker plots, cell populations were compared between clinical groups by Kruskal-Wallis test with Dunn's correction for multiple comparisons for each cell differentiation subtype, with only significant (*P* < 0.05) results shown. The colour under each cell phenotype bar chart shows its representation in the pie charts. Data are from C (*n* = 56), MS (*n* = 46), ME-M (*n* = 120), and ME-S (*n* = 21). ^*^P < 0.05, ^**^P < 0.01, ^***^P < 0.001, ^****^P < 0.0001.

More detailed phenotyping of the circulating T cell population revealed no major differences in the distribution of CD45RA- and CCR7-defined naïve and memory T cell populations in univariate analysis (Figure 4). In a more detailed phenotypic analysis of CD4^+^ and CD8^+^ T cell compartments by analysis of CD28 and CD57 alongside CCR7 and CD45RA, we found that there were no differences in the CD4^+^ T cell differentiation subpopulations in people with ME/CFS, although there were altered cell population frequencies in people with MS (Figure 5A). However, modest differences were observed in several differentiation populations in the CD8^+^ T cell compartment (Figure 5B) with a small increase in the proportion of EM cells (CCR7-CD45RA-CD28-CD57-) and a reduction in the proportion of terminally differentiated cytotoxic effector TEMRA cells (CCR7-CD45RA+CD28–CD57+) in people living with ME/CFS. There were also small changes in other minor CD8+ T cell populations, with small reductions in proportions of CCR7-CD45RA+CD28+CD57+, CCR7+CD45RA-CD28+CD57-, and CCR7-CD45RA-CD28+CD57- cells in people with ME/CFS and an increased proportion of CCR7+CD45RA+CD28-CD57- cells.

**Figure 6:**
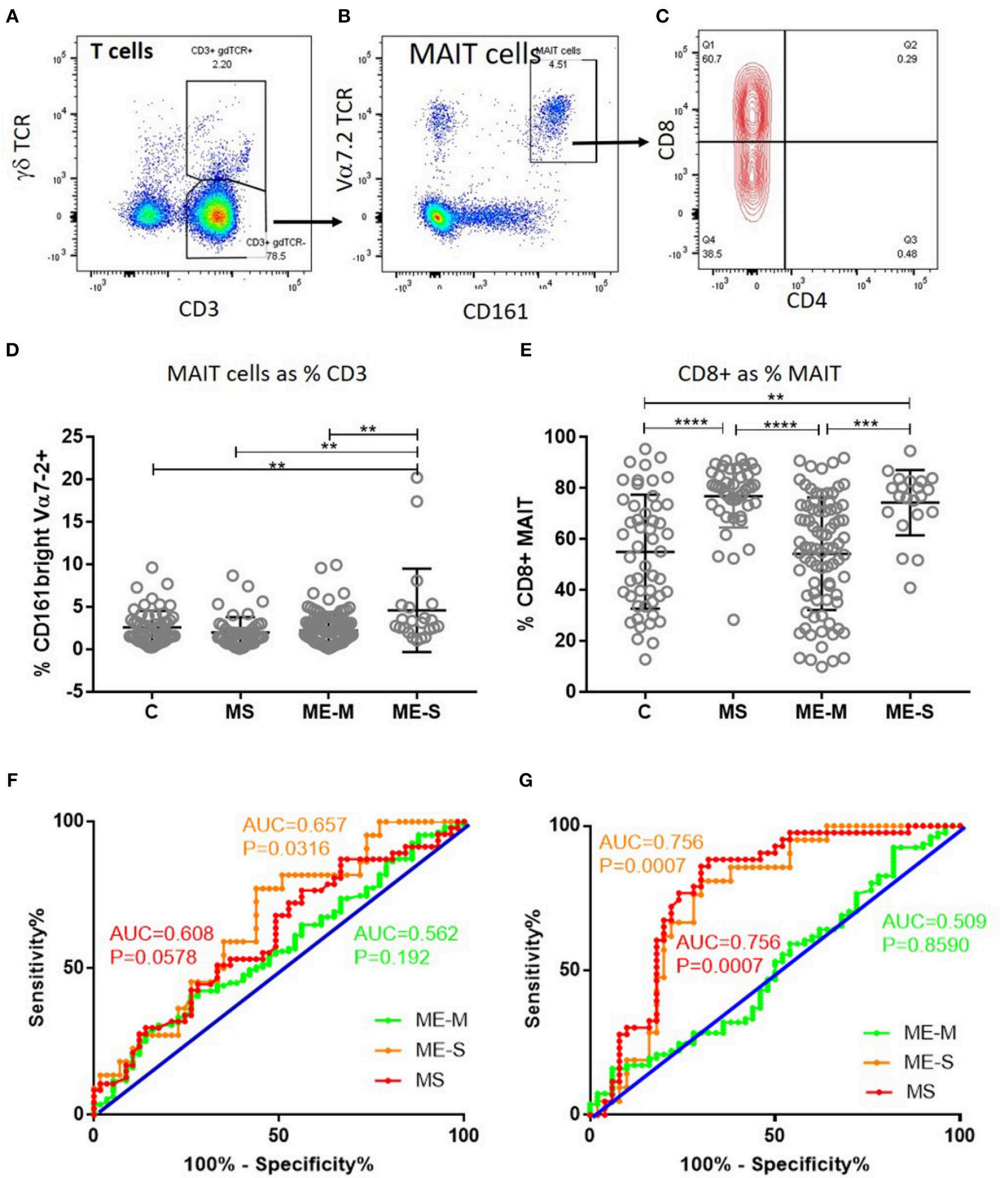
Mucosal Associated Invariant T cells in peripheral blood of people with ME/CFS, people with MS and healthy controls. By flow cytometry, within the CD3^+^γδTCR^−^ T cell population **(A)**, MAIT cells were identified as Vα7.2^+^CD161^++^ in PBMC **(B)**. MAIT cells were further characterised based on CD4^+^ or CD8^+^ expression **(C)**. **(D)** The proportion of T cells which were MAIT cells in PBMC from mild-moderate ME/CFS (ME-M), severely affected ME/CFS (ME-S), multiple sclerosis (MS), and healthy controls **(C)** is shown. **(E)** The proportion of MAIT cells which were CD8^+^ is shown for each individual. **(A–C)** Data from one representative severely affected ME/CFS patient. **(D,E)** Data are from C (*n* = 56), MS (*n* = 46), ME-M (*n* = 120), and ME-S (*n* = 21). Clinical groups were compared by Kruskal-Wallis test for non-parametric data: ^**^P < 0.01, ^***^P < 0.001, ^****^P < 0.0001. **(F)** ROC curve analysis of proportions of the CD3^+^γδTCR^−^ T cell population which were MAIT cells for the different patient groups relative to the Healthy Controls. **(G)** ROC curve analysis of proportions of MAIT cells which were CD8+ for the different patient groups relative to the Healthy Controls. AUC, Area Under the Curve.

Of greater potential relevance, however, using TCR Vα7.2 and CD161 to define circulating populations of mucosal associated invariant T (MAIT) cells, we observed highly significant differences between the groups in the proportion of T cells that are MAIT cells and the proportion of MAIT cells that express CD8 (unadjusted ANOVA, *P* < 0.001 in both cases) (Figure 6). After adjusting for confounding, the multiple linear regression analysis revealed that this was due to increased proportions of MAIT cells (*P* < 0.001), and particularly of CD8^+^ MAIT cells, in people with severe ME/CFS compared to healthy controls (*P* < 10^−5^). Importantly however, although the overall proportions of MAIT cells were not higher among people with MS than among healthy controls, their MAIT cells were also heavily skewed to the CD8^+^ subset, indicating that this is not a diagnostic feature of severe ME/CFS cases. ROC analysis showed that the total MAIT cell frequency in the T cell population (Figure 6F) showed a weak capacity to discriminate severely affected ME/CFS patients from healthy controls, but that the mild/moderately affected patients were not predicted by this measure. Potentially more importantly, the proportion of MAIT cells which were CD8^+^ (Figure 6G) had a greater capacity to discriminate severe ME/CFS from health, with an AUC of 0.756. In accordance with previous reports (38), the vast majority of MAIT cells in all subjects had an undifferentiated (CD28^+^ CD57^−^) phenotype (Supplementary Figure S3).

**Figure 7:**
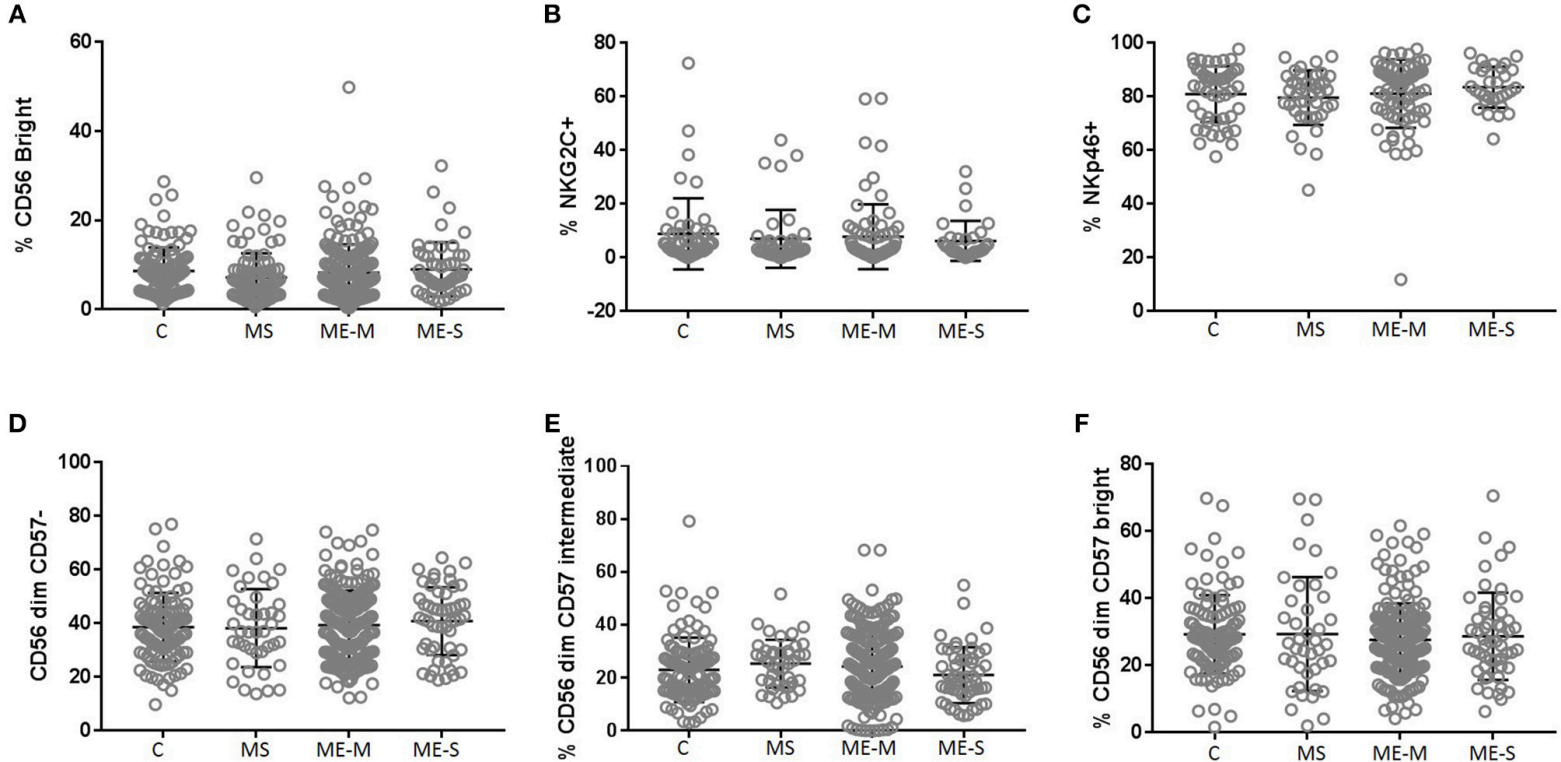
Proportions of Natural Killer Cell subsets in PBMC from people with ME/CFS, people with MS and healthy controls. By flow cytometry, NK cells were defined as CD3^−^CD56^+^ as shown in Figure 1. Subpopulations were defined as CD56^bright^ **(A)**, NKG2C^+^ **(B)**, NKp46^+^**(C)**, CD56^dim^CD57^−^ **(D)**^−^, CD56^dim^CD57^intermediate^**(E)**, or CD56^dim^CD57^bright^**(F)** for PBMC from mild/moderately affected ME/CFS (ME-M) or severely affected ME/CFS (ME-S), multiple sclerosis (MS) patients or healthy control individuals **(C)**. Populations of each type of NK cell were compared across clinical groups by ANOVA, but no significant differences were observed (*P* < 0.05). The bars show the Mean ± SD. Data are from healthy controls (C, *n* = 107), multiple sclerosis (MS: *n* = 46), mild/moderate ME/CFS (ME-M: *n* = 197), and severely affected ME/CFS (ME-S: *n* = 54) for **(A,D–F)** and from C (*n* = 50), MS (*n* = 41), ME-M (*n* = 76), and ME-S (*n* = 32) for **(B,C)**.

Looking in more detail at the phenotype of the NK cell population, we observed no significant differences in the proportions of CD56 bright cells, in the distribution of CD57^−^, CD57^+^ or CD57^++^ cells or in the proportions of cells expressing either NKG2C or NKp46 (Figure 7) or in the expression of six different killer cell immunoglobulin-like receptors (KIR) (Supplementary Figure S4) in either the adjusted or the unadjusted analysis.

### T Cell and NK Cell Function

**Figure 8:**
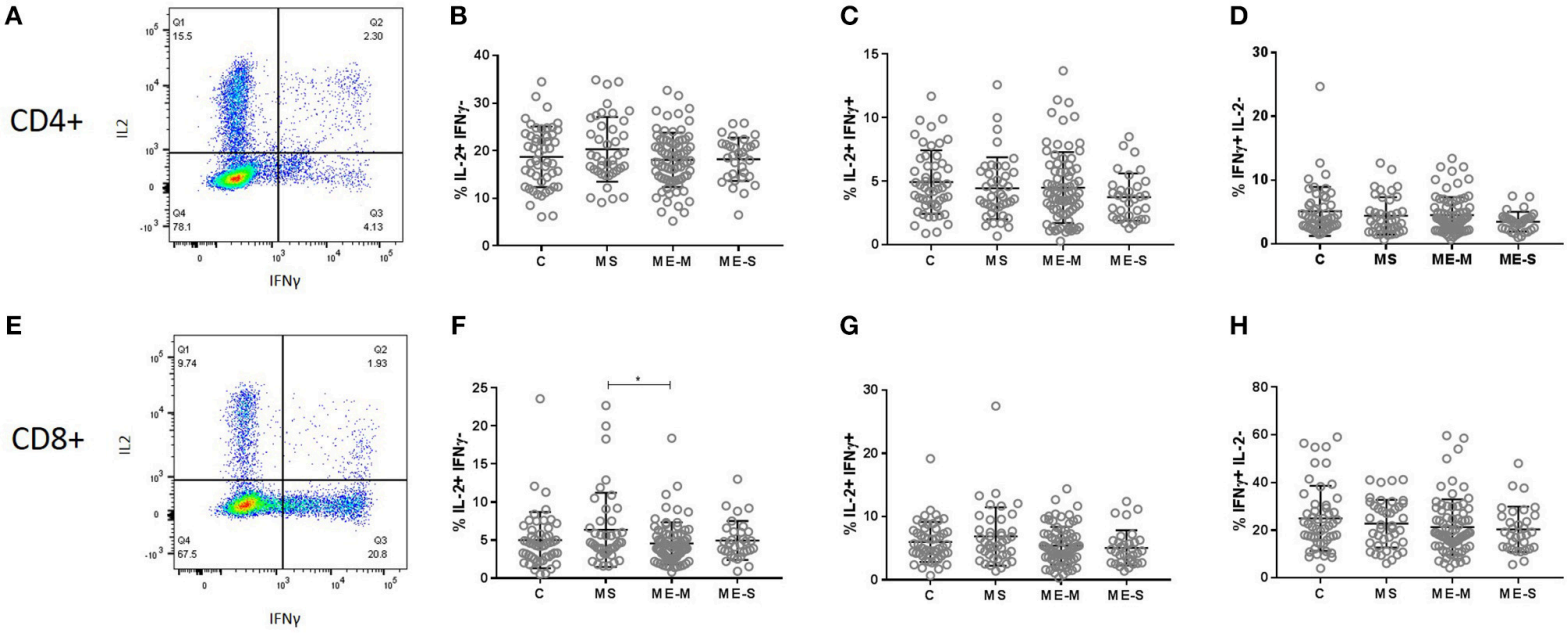
Production of cytokines by T cells in response to stimulation *in vitro*. PBMC from mild/moderately affected ME/CFS (ME-M: *n* = 76), severely affected ME/CFS (ME-S: *n* = 32) or multiple sclerosis (MS: *n* = 41) patients or healthy control (C: *n* = 50) individuals were cultured *in vitro* with PMA and ionomycin for 4 h. The production of IFNγ and IL-2 cytokines was assessed in CD4^+^ **(A–D)** and CD8^+^ **(E–H)** T cells, with the proportions of cells which produced only IL2 **(B,F)**, both IL2 and IFNγ **(C,G)** or only IFNγ **(D,H)** calculated for each study participant. Within the dot plots, the lines show the means and the error bars show ± SD.

We next compared the ability of T cells and NK cells from healthy controls, people with MS and PWME to respond to *in vitro* stimulation. For assessment of T cell responsiveness, PBMCs were cultured for 4 h with the mitogenic cocktail of PMA and ionomycin and then stained for intracellular cytokines. The proportion of cells producing IL-2, IFN-γ or a combination of IL-2 and IFN-γ was assessed in the CD3^+^ CD4^+^ and the CD3^+^ CD8^+^ cell populations but no significant differences were observed among the groups in either the adjusted or unadjusted analysis (Figure 8).

**Figure 9:**
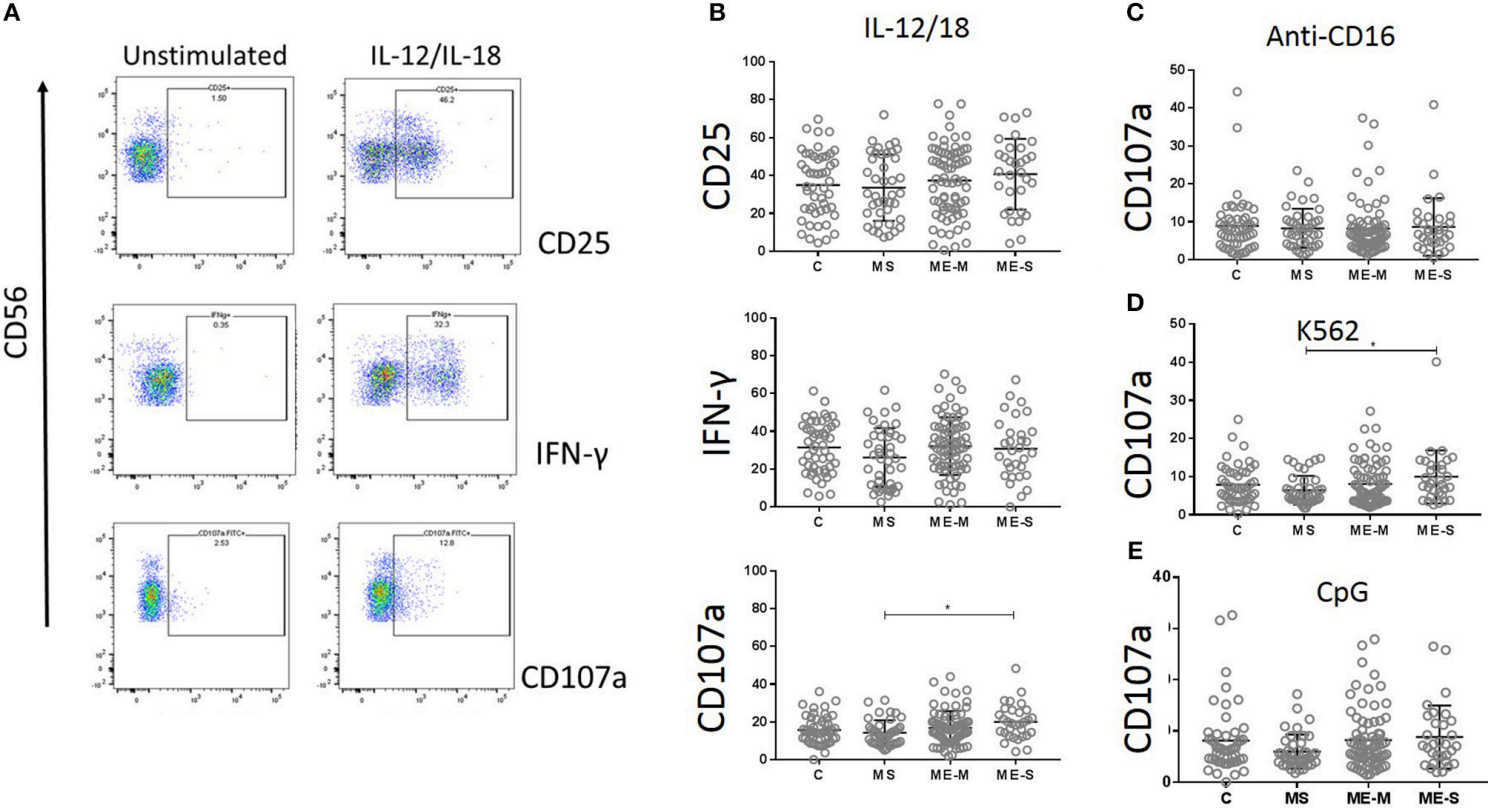
Natural Killer Cell function *in vitro* in people with ME/CFS, people with MS and healthy controls. PBMC were incubated *in vitro* various stimuli and NK cell function analysed by flow cytometry. **(A)** Flow cytometry gating strategy showing NK cell (CD3^−^ CD56^+^) expression of CD25, IFNγ or CD107a (degranulation marker) following incubation with or without IL12/IL18 for 18 h, in one representative donor. **(B)** Expression of CD25, IFNγ, and CD107 in NK cells following IL12/IL18 stimulation of PBMC from mild/moderately affected ME/CFS (ME-M: *n* = 76), severely affected ME/CFS (ME-S: *n* = 32) or multiple sclerosis (MS: *n* = 41) patients or healthy control (C: *n* = 50) individuals. CD107a expression on NK cells following 18 h stimulation by **(C)** cross-linking anti-CD16 monoclonal antibodies, **(D)** MHC Class-I deficient K562 cells or **(E)** CpG. Within the dot plots, the lines show the means and the error bars show ± SD.

NK cell function was assessed by culturing PBMCs for 18 h with IL-12 plus IL-18, with CpG, with MHC Class-I deficient K562 cells or in plates coated with a cross-linking antibody to CD16. CD3^−^/CD56^+^ NK cells were gated and analysed for intracellular IFN-γ or for cell surface expression of the activation marker CD25 or the degranulation marker CD107a (LAMP-1) (Figure 9). Again, no significant differences were detected between participants with ME/CFS and controls in any of the responses.

### Interaction Between Herpesvirus Infection Status and Lymphocyte Phenotype and Function

Herpesvirus infection, most notably infection with CMV, is known to affect both the maturational phenotype and the function of human CD8^+^ T cells (39) and NK cells (28). We therefore compared lymphocyte phenotype and function among those subjects who were either seropositive or not for CMV. Across all the study groups, participants who were CMV-seropositive had significantly lower proportions of CD4^+^ and higher proportions of CD8^+^ T cells amongst their PBMC (Supplementary Figure S5). Following stimulation with PMA and ionomycin, higher proportions of both CD4^+^ and CD8^+^ T cells made IFNγ, and more cells were IL-2 and IFNγ double positive, amongst CMV seropositive individuals. The proportion of PBMC which were CD3^+^CD56^+^ (NKT-like) cells was highly significantly enhanced amongst CMV-positive participants. In the NK cell population, more cells were NKG2C^+^, and there were decreased proportions of CD56^dim^CD57^intermediate^ and increased proportions of CD56^dim^CD57^bright^ amongst CMV-seropositive participants. Amongst CMV-seropositive individuals, there was a decreased *in vitro* response to IL-12 and IL-18 stimulation, with significantly reduced CD25, CD107, and IFNγ expression (Supplementary Figure S5G).

## Discussion

In a cohort of 250 well-characterised people living with ME/CFS, including a number who are severely affected, we have identified differences in peripheral immune cell phenotype compared to healthy control individuals. These include highly significantly enhanced proportions of mucosal associated invariant T cells, particularly CD8^+^ MAIT cells, in PWME compared to healthy controls, and these were further enhanced in individuals who had severe disease manifestation. We also found an enhancement in the proportion of effector memory CD8^+^ T cells, a reduction in the proportion of terminally differentiated effector TEMRA cells, and minor modulations in different CD8^+^ T cell differentiation subcompartments in PWME. The functional role and consequence of change within these different CD8^+^ T cell subpopulations remains to be determined. We did not find any evidence for altered function of T cells in PWME. Of note, we did not find any differences in the proportions of NK cells in PWME, or in their differentiation status, their expression of KIR receptors or of activation markers *ex vivo* or following *in vitro* stimulation. Based on previous reports of abnormal NK cell function in ME/CFS (17–19, 24, 25, 27, 40) this was an unexpected finding, although is consistent with a small number of previous reports of normal NK cell proportion and function (21–23). In our system, we cultured PBMC for 18 h to allow time for direct and indirect cell activation: it is possible that differences in degranulation or cytokine production may have been observed with a shorter stimulation period. However, we did find significant differences in NK cell differentiation status and cytokine production in study participants across all clinical groups who were CMV-positive, detectable after the 18 h culture period. CMV has a dramatic effect on NK cells (28) and it is possible that undetermined CMV status was a confounding factor underlying previous reports of NK cell dysfunction in ME/CFS. Importantly, the differences we observed in MAIT cells and CD8^+^ T cells in PWME were consistent when CMV serostatus was adjusted for.

ME/CFS is often considered to be triggered by an acute viral infection, and various herpes virus family members have been implicated (41). Here, we found no difference in the prevalence of seropositivity for six herpes viruses (CMV, EBV, HSV-1, HSV-2, VZV, HHV6) between PWME and healthy controls, nor in the concentration of anti-virus antibodies present. Nonetheless, the possibility remains that herpes viruses may be important in ME/CFS pathogenesis, that virus reactivation may trigger a worsening of symptoms, and that measurement of antibody titres to alternative viral antigens might provide a more relevant measure (42). Thus, although the seroprevalence of EBV did not differ among the MS patients, PWME or healthy controls, MS patients did have significantly higher titres of antibodies to EBV Viral Capsid Antigen, in line with previous reports [reviewed by Burnard et al. (43)] and potentially implying recent virus reactivation.

The increased frequency of CD8^+^ MAIT cells in those severely affected by ME/CFS observed here has not previously been reported. Notably, a small number of severely affected ME/CFS patients had exceedingly high frequencies of these cells. Peripheral MAIT cell frequencies in healthy volunteers have been reported to increase over 2-fold following acute exercise (44), suggesting that the peripheral immune phenotype at rest in some PWME resembles a post-exercise state. The more remarkable finding, however, was the overwhelming predominance of CD8^+^ cells among the MAIT cell population in severely affected PWME. Moreover, although overall frequencies of MAIT cells did not differ between MS patients and healthy controls, their MAIT cells were also highly enriched for CD8^+^ cells, confirming a previous report of high frequencies of CD8^+^ CD161^+^ T cells in MS patients (45). MAIT cells are innate-like T cells which recognise vitamin B-related antigens from microbes presented in the context of the MHC Class I-related molecule MR1 (46), and can also respond to cytokine stimulation. CD8^+^ MAIT cells are able to kill infected cells via perforin/granzyme secretion and therefore have a protective role in microbial infection (47), but they have recently been implicated in the pathology of various non-infectious diseases such as cancer (48), colitis (49) and autoimmune diseases such as rheumatoid arthritis (50) and type 1 diabetes (51). In MS, these cells are described as pro-inflammatory and pathogenic. It is possible that the increase in MAIT cell frequency in some PWME is driven by changes in the gut microbiome (52) or alternatively could be related to other, as yet uncharacterised, changes in the immune system. In future studies, the functional phenotype of these CD8^+^ MAIT cells in PWME could be ascertained, to determine if they may be contributing to disease pathology. The gut microbiome in ME/CFS should also be investigated further. Eventually a biomarker signature might be developed for diagnosis of ME/CFS: in this context the proportion of CD8^+^ MAIT cells in peripheral blood had modest discriminatory capacity alone, but might contribute to a combined factor signature. In this regard, testing of candidate biomarker signatures in large, independent, longitudinal validation cohorts will be vital, especially considering historically mixed reports of biological phenotype in ME/CFS.

The reason for the altered frequencies of various intermediately differentiated CD8^+^ T cells in ME/CFS is unclear, and is likely to be functionally linked with the concomitant reduction in effector memory CD8^+^ T cells (53). As there was no difference in the frequency of naïve or terminally differentiated CD8^+^ T cells, it is possible that in ME/CFS the cells are rapidly driven through this intermediate stage to terminal differentiation and are then lost by cell death. The study design did not allow us to determine the interaction between the enhanced MAIT cell compartment and the altered overall T cell differentiation status: this can be addressed in future studies. The driver behind the faster transition towards terminal differentiation could be ongoing antigenic stimulation, possibly due to persistent viral infection or autoimmunity. The reduced effector memory T cell populations in PWME may in part explain the increased susceptibility to infection which is commonly reported in ME/CFS.

The proportions of CD4^+^ and CD8^+^ T cells were normal in PWME. However, in MS patients there was a significant skew towards increased proportions of CD4^+^ T cells, lower CD8^+^ T cells and therefore a raised CD4^+^/CD8^+^ T cell ratio. Previously, there have been mixed reports in PWME, with increased (54) or decreased (55, 56) CD4^+^/CD8^+^ T cell ratios observed, although heterogeneity is commonly reported and may be confounded by CMV infection status.

In summary, in a large cohort of very carefully phenotyped people living with ME/CFS, we have observed significant differences in peripheral T lymphocyte phenotypes but have been unable to confirm previous reports of distorted NK cell phenotype or function. Importantly, we excluded from this study individuals with evidence of ongoing acute infection, those with obvious comorbidities and those with a history of taking immunomodulatory medications. We have also confirmed that CMV seropositivity has a major impact on the phenotype and function of NK cells and T cells, underlining the paramount importance of assessing CMV and other herpes virus infection status in studies of human immune status. Any of these factors may have confounded the analysis of previous studies. The most striking finding of this study, the increased proportion of circulating MAIT cells in people severely affected by ME/CFS, and the very high proportion of CD8^+^ MAIT cells in severely affected PWME and in MS patients, warrants further investigation. This observation also underlines the importance of including non-fatigued individuals with other incapacitating illnesses (such as MS patients) in studies of ME/CFS to control for the physiological effects of reduced levels of physical activity. Although one might debate whether MS patients are the most appropriate such group, the very large immunological database for MS does facilitate benchmarking of observations and may provide insight into disease pathogenesis pathways in ME/CFS by comparison with a more extensively documented disease.

## Data Availability

The datasets generated for this study are available on request to the corresponding author.

## Ethics Statement

This study was carried out in accordance with the recommendations of The London School of Hygiene & Tropical Medicine (LSHTM) Research Ethics Committee (Ref. 6123) and the National Research Ethics Service (NRES) London-Bloomsbury Research Ethics Committee (REC ref. 11/10/1760, IRAS ID: 77765), with written informed consent from all subjects. All subjects gave written informed consent in accordance with the Declaration of Helsinki. The protocol was approved by the LSHTM Research Ethics Committee and the NRES London-Bloomsbury Research Ethics Committee.

## Author Contributions

JC, HD, LN, EL, and ER devised the study and obtained funding. EL, EB, LN, and CK designed and implemented the clinical study. CK conducted the clinical assessments and collected samples. JC, HD, and ER designed and implemented the laboratory studies. EK, J-SL and A-SW conducted the laboratory assays. JC, EK, NS, EL, and ER analysed the data. JC, NS, LN, EL, and ER wrote the manuscript. All authors reviewed and approved the final draft of the manuscript.

### Conflict of Interest Statement

The authors declare that the research was conducted in the absence of any commercial or financial relationships that could be construed as a potential conflict of interest.
