# Supplementary material for: Cellular Immune Function in Myalgic Encephalomyelitis/Chronic Fatigue Syndrome (ME/CFS)
Source: Front Immunol. 2019 Apr 16;10:796. doi: 10.3389/fimmu.2019.00796 (PMC6477089; doi:10.3389/fimmu.2019.00796)
Supplement: Supplementary file 1 [file Table_1.pdf]

Supplementary Table S1: Clinical parameters collected from study population

Questionnaires	Clinical assessments	Blood tests
• Symptoms experienced	• urinalysis by dipstick	• full blood count
• Sociodemographic variables	• pulse oximetry	• blood chemistry and creatinine
• Family and individual health histories	• blood pressure (seated and standing)	• liver function
• Potential risk factors (exposures)	• standing height	• thyroid function
• Medical Outcomes Survey Short Form (SF-36v2™)	• weight and bioimpedance	• C reactive protein (CRP)
• General Health Questionnaire (GHQ-28)	• waist circumference	• erythrocyte sedimentation rate (ESR)
• Epworth sleepiness score	• hand grip strength test	• rheumatoid factor
• Fatigue scales assessing severity and disability	• spirometry	• tissue transglutaminase antibodies
• Pain and fatigue analogue scale		• serum vitamin B12
		• folate
