# Supplementary material for: Cellular Immune Function in Myalgic Encephalomyelitis/Chronic Fatigue Syndrome (ME/CFS)
Source: Front Immunol. 2019 Apr 16;10:796. doi: 10.3389/fimmu.2019.00796 (PMC6477089; doi:10.3389/fimmu.2019.00796)
Supplement: Supplementary file 3 [file Table_3.pdf]

Supplementary Table S3: Flow cytometry staining panels for differentiation status and MAIT cell phenotype

Panel A					Panel B				
**Marker**	**Fluorochrome**	**Clone**	**Company**	**Cat#**	**Marker**	**Fluorochrome**	**Clone**	**Company**	**Cat#**
CD45	FITC	2D1	BD	345808	CD45RA	BB515	H100	BD	564552
CD11c	PE	B-ly6	BD	555392	CD161	PE	191B8	Miltenyi	130-092-677
CD123	PE-eF610	6H6	eBio	61-1239	Va7.2	PE-DAZZLE	3C10	Biolegend	351730
CD19	PC5.5	HIB19	eBio	45-0199	CD28	PC5.5	CD28.2	eBio	45-0289
Va24	PC7	6B11	Biolegend	342911	gdTCR	PC7	B1	Biolegend	331221
Vb11	APC	REA657	Miltenyi	130109-	CCR7	APC	3D12	eBio	17-1979
CD14	AF700	61D3	eBio	56-0149-	CD4	AF700	RPA-T4	eBio	56-0049
Viability	eF780	n/a	eBio	65-0865-14	Viability	eF780	n/a	eBio	65-0865-14
CD57	e450	TB01	eBio	48-0577-	CD57	e450	TB01	eBio	48-0577-
CD3	V500	UCHT1	BD	561416	CD3	V500	UCHT1	BD	561416
CD56	BV605	HCD56	Biolegend	318334	CD8	BV605	RPA-T8	Biolegend	301040
