# Supplementary figures and images for: Cellular Immune Function in Myalgic Encephalomyelitis/Chronic Fatigue Syndrome (ME/CFS)
Source: Front Immunol. 2019 Apr 16;10:796. doi: 10.3389/fimmu.2019.00796 (PMC6477089; doi:10.3389/fimmu.2019.00796)

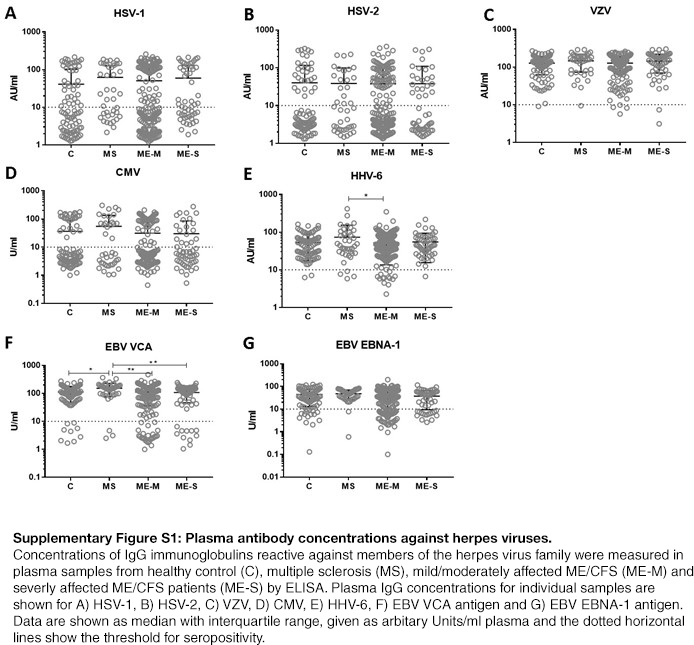

Supplement: Supplementary file 4 [file Image_1.jpeg]

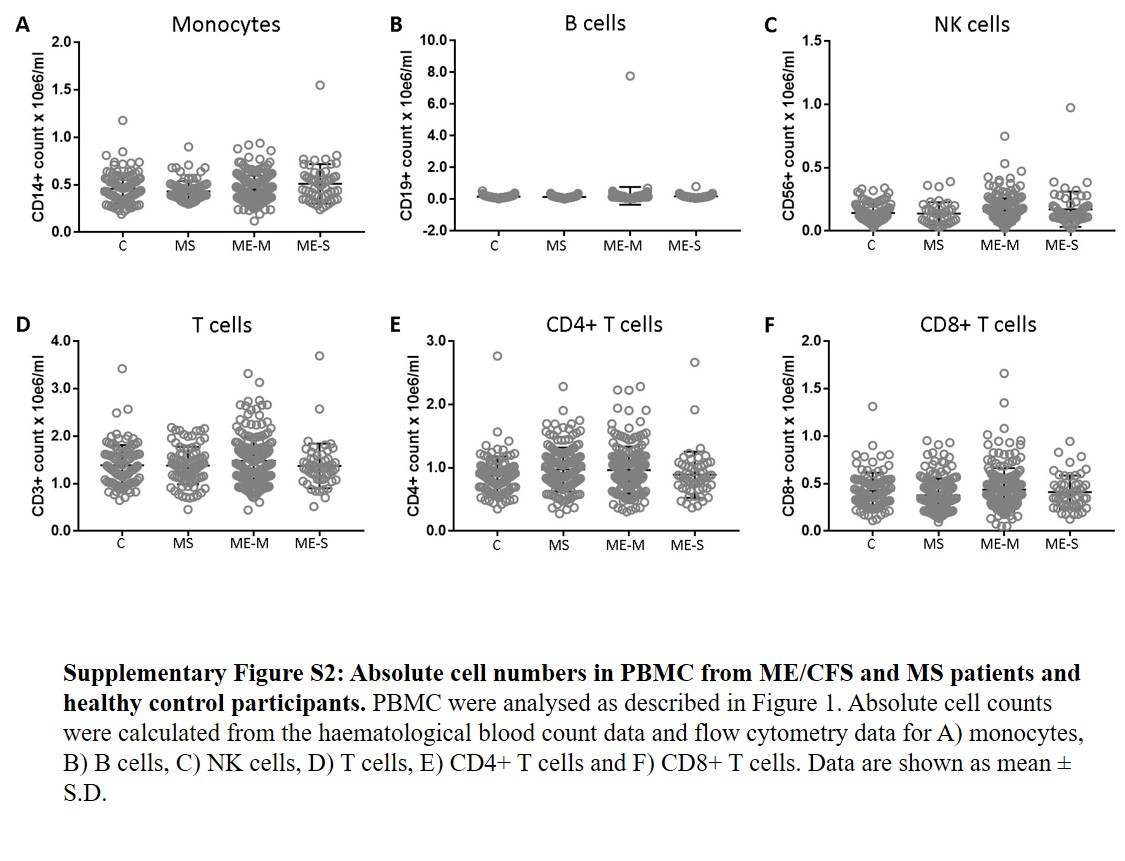

Supplement: Supplementary file 5 [file Image_2.jpeg]

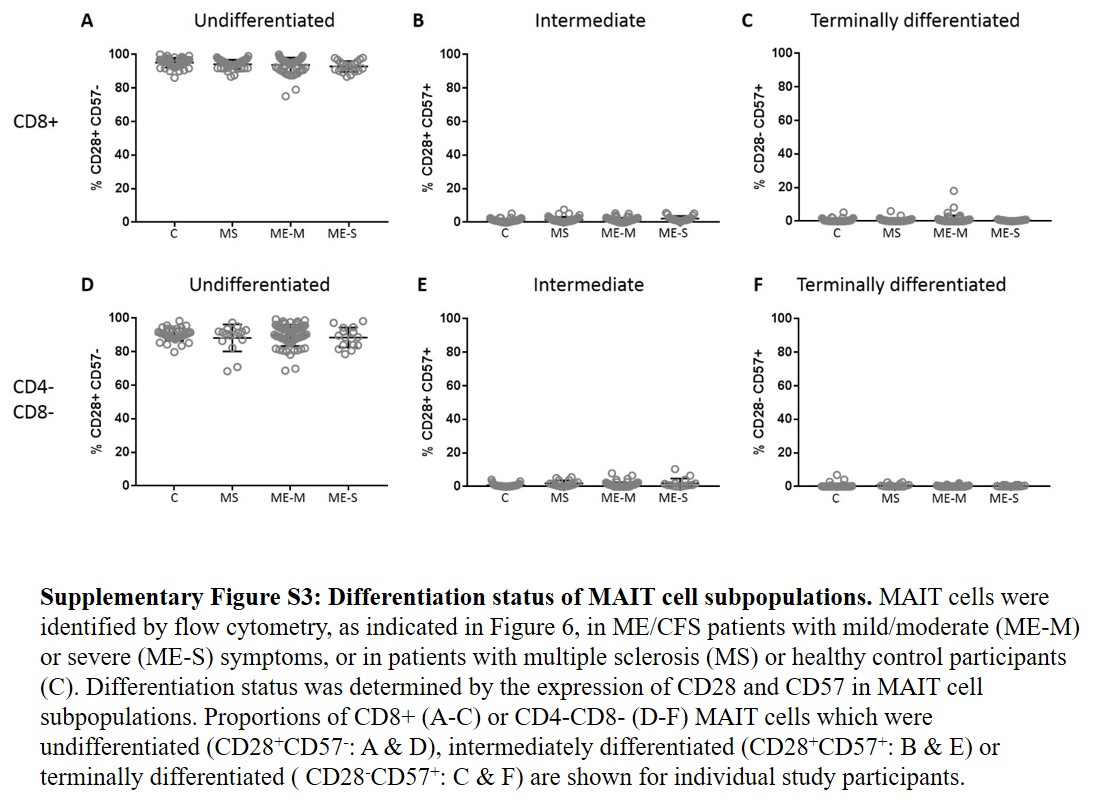

Supplement: Supplementary file 6 [file Image_3.jpeg]

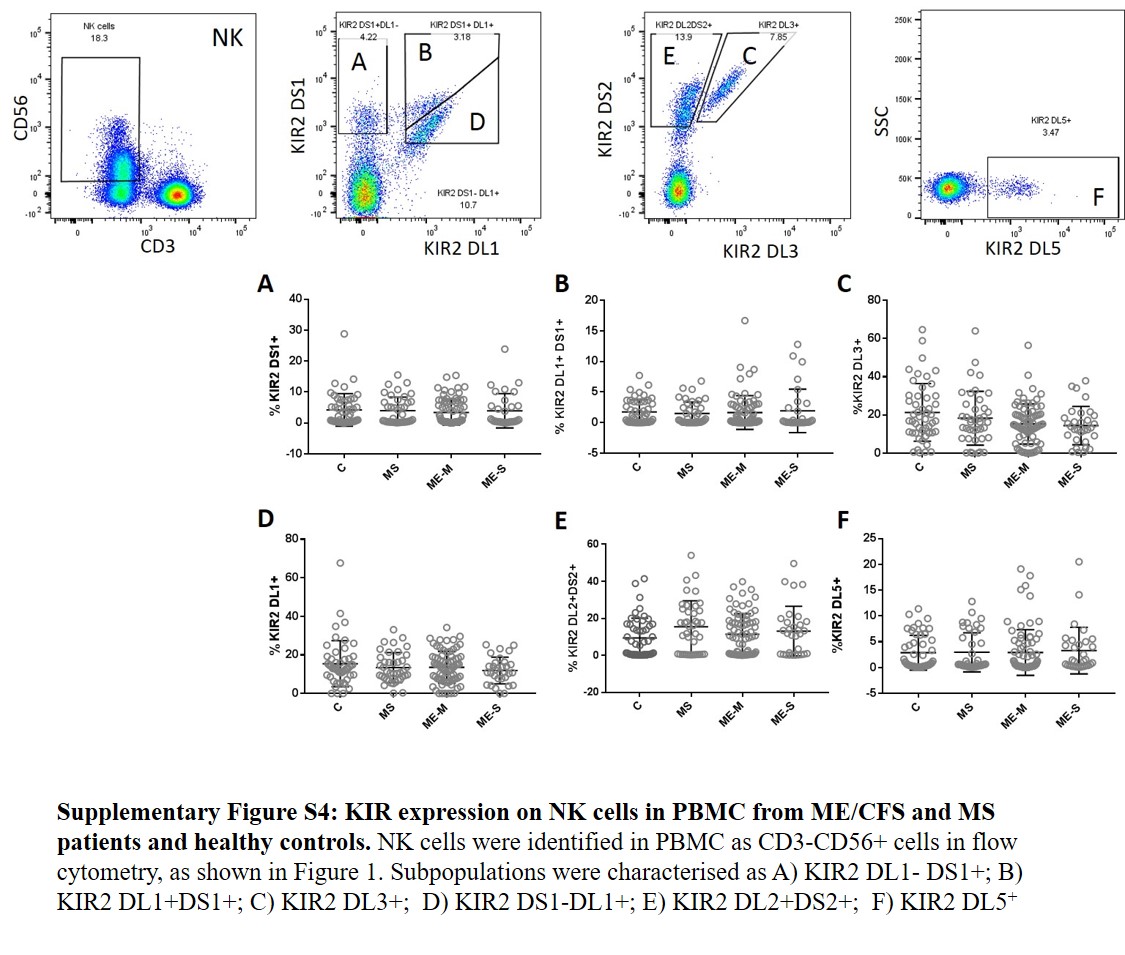

Supplement: Supplementary file 7 [file Image_4.jpeg]

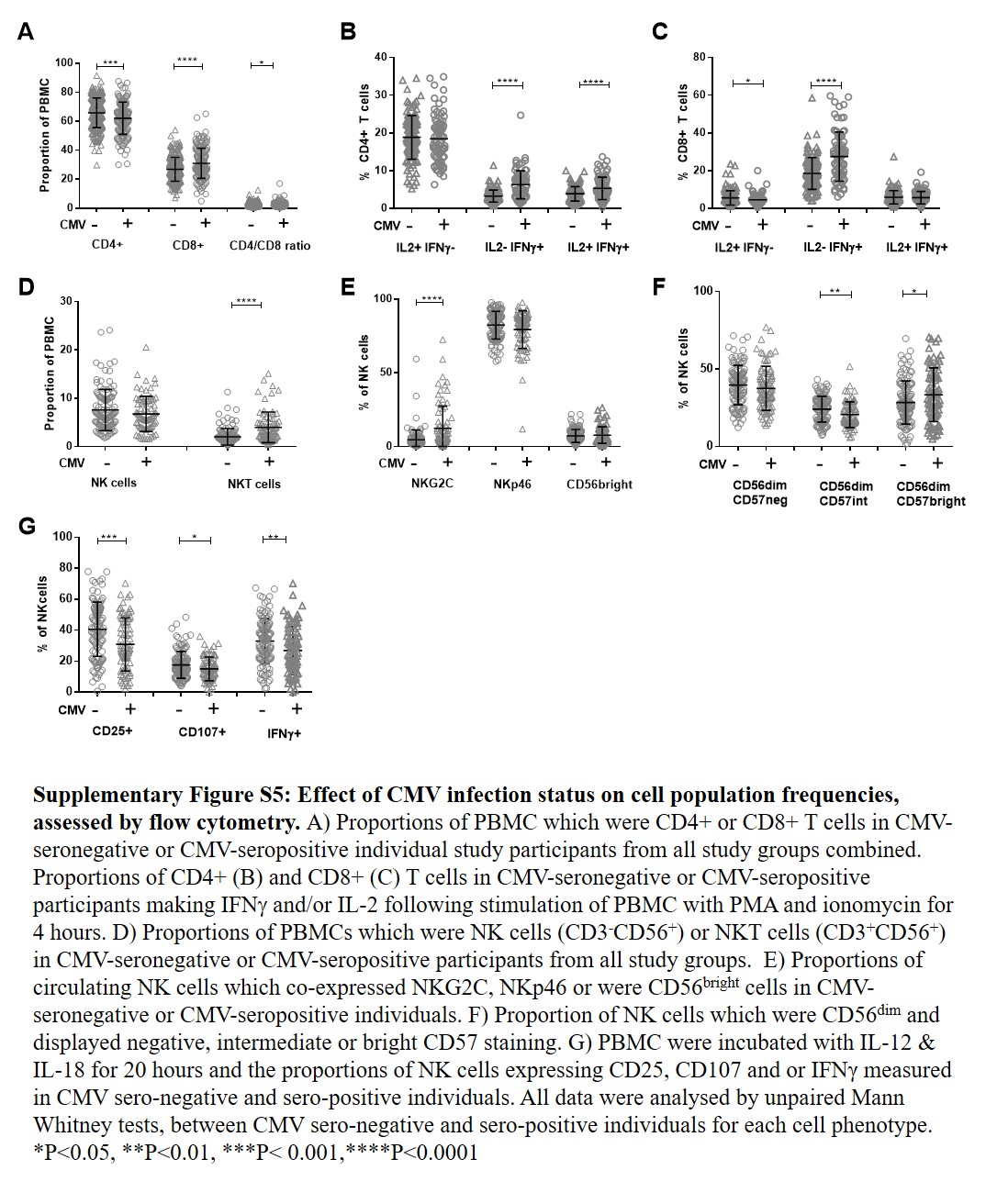

Supplement: Supplementary file 8 [file Image_5.jpeg]
