# Supplementary material for: Cellular Immune Function in Myalgic Encephalomyelitis/Chronic Fatigue Syndrome (ME/CFS)
Source: Front Immunol. 2019 Apr 16;10:796. doi: 10.3389/fimmu.2019.00796 (PMC6477089; doi:10.3389/fimmu.2019.00796)
Supplement: Supplementary file 2 [file Table_2.pdf]

Supplementary Table S2: Flow cytometry staining panels for overall leukocyte and for NK cell phenotype analysis

Leucocyte/ Tcell Panel					KIR Panel					NK Culture panel				
Marker	Fluorochr	Clone	Company	Cat#	Marker	Fluorochr	Clone	Company	Cat#	Marker	Fluorochr	Clone	Company	Cat#
CD8	PE-Cy5	RPA-T8	eBioscience	15-0088	KIR2 DL3	FITC	REA147	Miltenyi	130-100	CD107a*	FITC	H4A3	BD	555800
CD19	PE-Cy7	HIB19	eBioscience	25-0199	KIR2 DL1/DS5	PE	143211	R&D	FAB1844P	NKG2C	PE	134591	R&D Systems	FAB138P
CD11c	APC	3.9	eBioscience	17-0116	KIR2 DL2/DS2/ DL3	PC5.5	GL183	BC	A66900	CD25	PerCP-Cy5.5	BC96	eBioscience	45-0259
CD14	APC-e780	61D3	eBioscience	47-0149	KIR2 DL1/S1	PE-Cy7	EB6B	BC	A66899	CD56	PE-Cy7	NCAM16.2	BD	335826
CD4	V450	RPA-T4	BD	560345	KIR2 DL5	APC	UP-R1	Miltenyi	130-098	NKp46	APC	9E2	eBioscience	17-3359
CD3	V500	UCHT1	BD	561416	CD56	APC-e780	NCAM16.2	eBioscience	47-0567	CD57	e450	TBO1	eBioscience	48-0577
IFNg	FITC	D9D10	ABDSerotec	MCA1581F						CD3	V500	UCHT1	BD	561416
IL-2	PE	MQ1-7H2	eBioscience	12-7029	CD3	V500	UCHT1	BD	561416	IFNγ	APC-e780	4S.B3	eBioscience	47-7319
